# Paroxysmal Complete Atrioventricular Block With Complete Left Bundle Branch Block Treated With Left Bundle Branch Area Pacing: A Case Report

**DOI:** 10.7759/cureus.75678

**Published:** 2024-12-13

**Authors:** Kiyotaka Tsuyuki, Naoto Nishina, Hiroshi Tsujimoto

**Affiliations:** 1 Cardiovascular Medicine, Soseikai General Hospital, Kyoto, JPN; 2 Clinical Engineering, Soseikai General Hospital, Kyoto, JPN

**Keywords:** cardiac resynchronization therapy (crt), conduction system pacing, left bundle branch area pacing, pace maker, pacing-induced cardiomyopathy

## Abstract

Left bundle branch area pacing (LBBAP) can effectively enhance cardiac contraction by engaging the conduction system. LBBAP, compared with right ventricular apex pacing, can reduce QRS duration and enhance left ventricular function. Consequently, LBBAP has been proposed as a viable alternative to cardiac resynchronization therapy (CRT).Here, we present a case illustrating the efficacy of the LBBAP in a patient with a complete atrioventricular block and complete left bundle branch block (CLBBB).

## Introduction

Complete atrioventricular block causes bradycardia due to dysfunction of the atrioventricular node and requires the implantation of a pacemaker. Evidence suggests that right ventricular pacing during pacemaker implantation contributes to an increased incidence of heart failure and atrial fibrillation [1]. Cardiac resynchronization therapy (CRT) has demonstrated efficacy in patients with an atrioventricular block requiring pacemaker implantation and concurrent left ventricular systolic dysfunction. CRT, compared to right ventricular pacing alone, has been shown to reduce mortality, heart failure exacerbation significantly, and left ventricular remodeling [2]. However, CRT is not indicated in cases of complete atrioventricular block with conventional left bundle branch block (CLBBB) and preserved left ventricular function, and left bundle branch area pacing (LBBAP) has emerged as a potential solution. LBBAP has been shown to shorten QRS duration and enhance left ventricular function by directly stimulating the left bundle branch via ventricular septal myocardial pacing compared with right ventricular apical pacing [3,4].

## Case presentation

**Figure 1:**
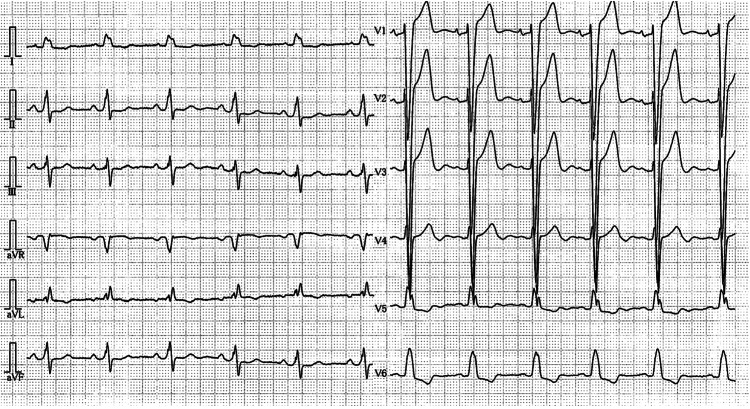
A 12-lead electrocardiogram revealed complete left bundle block in paroxysmal atrioventricular block.

**Figure 2:**
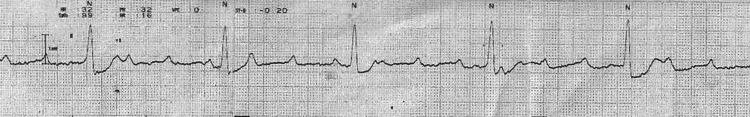
An ECG showed paroxysmal atrioventricular block.

A man in his 70s was admitted to our hospital with syncope. The medical history included cerebral infarction and a clopidogrel regimen. Although he had a history of cerebral infarction, he showed no obvious aftereffects such as quadriplegia or dysarthria. An electrocardiogram (ECG) revealed left bundle branch block (LBBB) and paroxysmal complete atrioventricular block, which was thought to be the cause of the syncope (Figures 1, 2).

**Figure 3:**
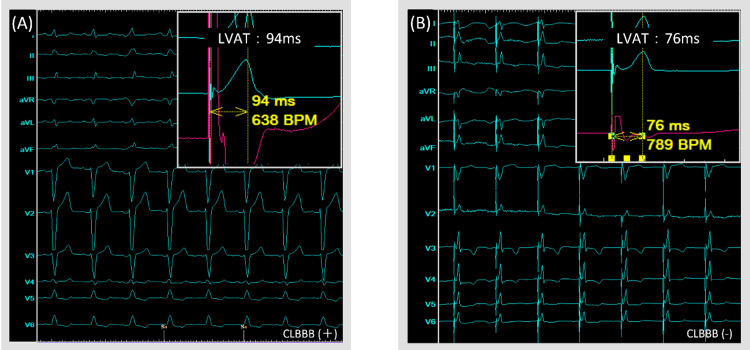
The ECG after implantation is shown. The disappearance of the LBBB on ECG and shortened LVAT from pacing spike to peak of the R-wave in lead V6 were noted. LBBB: left bundle branch block; LVAT: left ventricular activation time

Chest radiography revealed mild pulmonary congestion and an elevated cardiothoracic ratio (CTR) of 59.2%. Echocardiography showed no abnormalities in the left ventricular wall motion, and the left ventricular ejection fraction was relatively maintained at around 50%. Emergency coronary angiography revealed no indication of ischemic heart disease, prompting the placement of a temporary pacemaker. Permanent pacemaker implantation is warranted for symptomatic complete paroxysmal atrioventricular blocks. ECG findings of the LBBB, echocardiographic evidence of septal flash, and two-dimensional speckle tracking confirming dyssynchrony led to an attempt of LBBAP. A ventricular lead (SelectSecure™ 3830; Medtronic, Dublin, Ireland) was introduced into the interventricular septum via a C315His delivery catheter (Medtronic). Subsequent confirmation of the ventricular electrode tip position was achieved using a contrast medium followed by screwing. The position of the lead tip was visualized using a contrast medium from the catheter tip, and the lead was placed while taking care to avoid any significant drop in lead impedance. The disappearance of the LBBB on ECG, reduction in lead impedance post-screwing, and shortened left ventricular activation time (LVAT) from pacing spike to peak of the R-wave in lead V6 were noted, indicating favorable thresholds (Figure 3).

**Figure 4:**
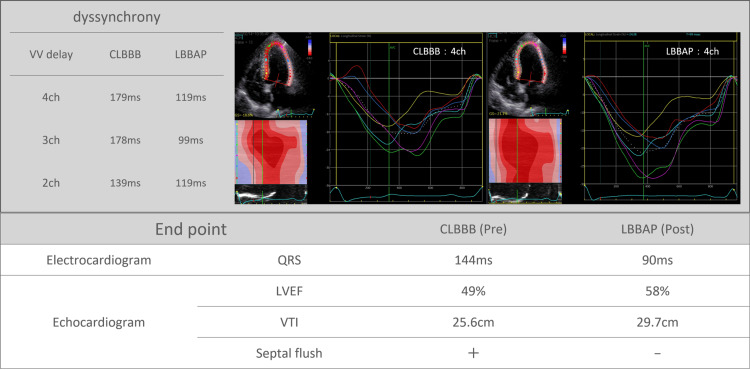
Speckle tracking analysis of mechanical dyssynchrony by echocardiography. LBBAP eliminated septal flash, improved dyssynchrony throughout ventricular activation phases. LVEF: left ventricular ejection fraction; VTI: velocity-time integral; CLBBB: conventional left bundle branch block; LBBAP: left bundle branch area pacing

The lead was connected to a pacemaker (Azure XT DR; Medtronic) and retained in situ. Echocardiography facilitated the assessment of the left ventricular ejection fraction (LVEF), velocity-time integral (VTI), septal flash, dyssynchrony changes, and QRS duration on ECG. LBBAP eliminated septal flash, improved dyssynchrony throughout ventricular activation phases, and enhanced VTI. ECG confirmed R waves from V2 and a 50-millisecond reduction in the QRS duration (Figure 4).

The patient was discharged on the 10th day after pacemaker implantation. Six months later, the pacing threshold remained stable, and the patient's condition was satisfactory.

## Discussion

The prevalence of pacing-induced cardiomyopathy (PICM) among patients with ventricular pacing dependence ranges from 10-20%, posing a significant challenge to pacemaker implantation [5]. However, PICM is infrequently observed in individuals with a QRS duration of 140 ms or less [6]. His bundle pacing, a form of intraventricular conduction system pacing, compared to right ventricular apical pacing, has been documented to better ameliorate the composite endpoint of heart failure and mortality [7]. Nevertheless, His bundle pacing is beset by challenges such as a heightened pacing threshold and procedural complexity.

LBBAP has emerged as a superior alternative to His bundle pacing, characterized by a lack of threshold elevation and comparable QRS width [8]. LBBAP is noted for its significantly shortened QRS duration compared with that associated with pacing at the right ventricular apex or septum, potentially averting the onset of PICM [9]. Additionally, it has been reported that LBBAP enhances myocardial synchrony in cases of conduction system disturbances such as CLBBB and holds promise for enhancing cardiac function [10]. In this case, LBBAP was performed for CLBBB, with electrocardiographic evidence revealing a diminished QRS width, echocardiography demonstrating the disappearance of the septal flash, and speckle tracking indicating enhanced synchrony. Successful LBBAP was determined based on established criteria, encompassing distinctive Qr, qR, or rSR patterns in lead V1 during unipolar pacing; a marked reduction in stimulus to LVAT exceeding 10ms, alterations in QRS duration and morphology during threshold assessment or programmed stimulation; LVAT of less than 80 ms, and a V6-V1 interpeak interval of 44 ms. Despite the absence of an R-wave in lead V1, meeting the criteria of VLAT < 80 ms and paced QRS < 120 ms led to LBBAP [11].

Contemporary guidelines advocate CRT for individuals with compromised left ventricular function and CLBBB. Traditionally, CRT has been implemented through biventricular pacing (BVP); however, a report suggested that LBBAP, compared to BVP, markedly diminishes mortality and heart failure-related hospitalizations [12]. Furthermore, in patients with heart failure and CLBBB, LBBAP has been reported to significantly reduce BNP and reduce the number of hospitalizations compared to BVP [10]. Reports on the efficacy and incidence of significant complications associated with LBBAP indicate a notable success rate of 80.5%, with no significant adverse events [13]. While LBBAP is yet to be delineated in the guidelines, its consideration based on individual cases is imperative for future clinical practice.

## Conclusions

In the current case, LBBAP was effective for a complete atrioventricular block with CLBBB. LBBAP presents a promising intervention for improving cardiac function in patients with CLBBB.
